# Reflections on 30 Years of AIDS

**DOI:** 10.3201/eid1706.100184

**Published:** 2011-06

**Authors:** Kevin M. De Cock, Harold W. Jaffe, James W. Curran

**Affiliations:** Author affiliations: Centers for Disease Control and Prevention, Atlanta, Georgia, USA (K.M. De Cock, H.W. Jaffe);; Emory University, Atlanta (J.W. Curran);; Emory Center for AIDS Research, Atlanta (J.W. Curran)

**Keywords:** HIV, AIDS, history, viruses, historical review

## Abstract

June 2011 marks the 30th anniversary of the first description of what became known as HIV/AIDS, now one of history’s worst pandemics. The basic public health tools of surveillance and epidemiologic investigation helped define the epidemic and led to initial prevention recommendations. Features of the epidemic, including the zoonotic origin of HIV and its spread through global travel, are central to the concept of emerging infectious diseases. As the epidemic expanded into developing countries, new models of global health and new global partnerships developed. Advocacy groups played a major role in mobilizing the response to the epidemic, having human rights as a central theme. Through the commitments of governments and private donors, modern HIV treatment has become available throughout the developing world. Although the end of the epidemic is not yet in sight and many challenges remain, the response has been remarkable and global health has changed for the better.

## Abstract 2

TOC summary: Although the end of the epidemic is not yet in sight, the remarkable response has improved health around the world.

We seem to think, with health problems as with other things, that science and technology will always save us, even though in the realm of human endeavor, it always comes (down) to people and our relationships—James Curran

One of the saddest days of my life was when my mother told me Superman did not exist…. “’cause you just thought… he always shows up and he saves all the good people… and I was crying because there was no one… coming with enough power to save us.”—Geoffrey Canada

On June 5, 1981, the Morbidity and Mortality Weekly Report (MMWR), published by the Centers for Disease Control and Prevention (CDC), described *Pneumocystis carinii* (now *P. jiroveci*) pneumonia in 5 homosexual men in Los Angeles, California, USA, documenting for the first time what became known as acquired immunodeficiency syndrome (AIDS). The accompanying editorial suggested that the illness might be related to the men’s sexual behavior. A month later, the MMWR reported additional diagnoses of *P. carinii* pneumonia, other opportunistic infections (OIs), and Kaposi sarcoma (KS) in homosexual men from New York City and California. These articles were sentinels for what became one of history’s worst pandemics, with >60 million infections, 30 million deaths, and no end in sight.

This 30th anniversary year of the first description of AIDS is also the 15th anniversary of the introduction of highly active antiretroviral therapy (ART). Henceforth, AIDS will have been a treatable condition longer than it was the inevitably fatal disease first recognized. We offer highlights and reflections from a predominantly global perspective on 3 decades of collective experience with AIDS.

## Early AIDS Surveillance and Epidemiology

To investigate this apparent outbreak, CDC investigators developed a simple surveillance case definition for what was first called KS/OI. The definition focused on certain OIs or KS in otherwise healthy persons and was used to establish a national reporting system. In light of new knowledge concerning AIDS and its underlying cause, the case definition was modified over time, but early surveillance indicated that an epidemic was under way and, in retrospect, had begun several years before the first reports. Retrospective testing of stored serum specimens from hepatitis patients in Los Angeles documented human immunodeficiency virus (HIV) infection as early as 1979.

The initial risk groups identified were men who have sex with men (MSM) and injection drug users (IDU). Field investigations and surveillance activities demonstrated sexually linked cases in MSM and in persons with hemophilia and transfusion recipients, implicating transmission by male-to-male sexual contact as well as through blood and blood products. Cases in heterosexual persons and infants indicated that transmission could also occur through heterosexual contact and from mother to child.

Within <2 years, the essential epidemiology of AIDS—groups at risk and modes of transmission—was established, although debate about transmission through blood and blood products continued for several months after CDC believed the evidence was clear. In March 1983, the US Public Health Service published the first recommendations for AIDS prevention, including a recommendation that members of risk groups limit their numbers of sex partners and not donate blood or plasma. Although these recommendations were made before the etiologic agent, HIV, had been identified, they initiated AIDS prevention efforts and have largely stood the test of time, as has promotion of condom use.

As evidence accumulated that AIDS would not be confined to MSM and IDU, media interest grew and fears of contagion increased. Fear about transmission through casual contact led to discrimination against persons with AIDS, including barring HIV-infected children from school. During this time, CDC was recognized as a source of trustworthy information, and the agency gained respect by placing science above political considerations. Having devoted 71 articles to AIDS during 1981–1985, MMWR played a central role in dissemination of health information for rational policy decisions.

CDC regularly consulted with the World Health Organization (WHO), which published global data in its Weekly Epidemiological Record. While cases of AIDS in MSM and IDU began to be reported from other countries, several European countries reported cases in black Africans with no history of drug use or male-to-male sex. In the United States, cases also occurred in recent migrants from Haiti, subsequently designated as a risk group. Although this designation was useful for public health purposes, it resulted in discrimination against Haitian Americans. The subsequent explanation for AIDS in Africans and Haitians without other risk factors was heterosexual transmission of the causative agent.

In 1983, HIV was discovered, an accomplishment for which French scientists received the Nobel Prize for Medicine in 2008. In 1985, a serologic test for HIV became commercially available. Agreement on HIV as the causative agent and the availability of a diagnostic test were closing features of these early years. Despite the potential for hysteria and some examples of irrational responses, science and reason prevailed; epidemiology and surveillance served as the foundation of society’s understanding and early response, as they would have to do repeatedly in future infectious disease epidemics.

## AIDS as a Metaphor for Emerging Infections and the New Global Health

Social and environmental change, increased public health awareness, and improved diagnostic tools led to the emergence and recognition of several new pathogens in the last third of the 20th century. After a prolonged period of complacency with regard to infectious diseases, in 1992 the Institute of Medicine published an influential report on emerging infectious diseases. This term referred to conditions that were increasing in incidence in human populations or threatening to do so, were newly introduced or detected, or were recognized as being linked to a chronic disease or syndrome. No agent and disease better exemplify this concept than HIV and AIDS.

HIV type 1, group M (HIV-1), the predominant cause of the AIDS epidemic, evolved from a virus that crossed the species barrier from chimpanzees to humans. The earliest retrospective diagnosis of HIV-1 infection was made from a serum specimen collected in 1959 in Kinshasa, capital of what is now the Democratic Republic of Congo. Two additional but rare groups of HIV-1 (N and O) cause related zoonotic infections that are essentially restricted to central Africa. HIV-2, a second type of HIV rarely found outside western Africa, originated in sooty mangabeys.

Phylogenetic analysis of HIV-1 and SIVcpz (the simian immunodeficiency virus of chimpanzees closely related to HIV-1), combined with knowledge about the geographic range of the chimpanzee host, *Pan troglodytes troglodytes*, suggest that this cross-species transmission took place in central Africa early in the 20th century. The exact circumstances of cross-species transmission in central Africa are uncertain, but opportunities for human exposure to simian viruses through hunting and related activities are abundant. Over time, the virus presumably adapted to the human host and began to spread from person to person. At some unknown point, it was introduced into the Western Hemisphere, including Haiti and the United States.

Although the epidemic appears to have begun in central Africa, HIV prevalence is now highest in southern Africa; the Republic of South Africa alone is home to about one sixth of the world’s HIV-infected persons. The reasons for this geographic distribution are not entirely clear, but biological factors, such as lack of male circumcision and rates of other genital (especially ulcerative) infections that facilitate HIV transmission, and social factors (some of which may have been influenced by the end of apartheid), such as frequent partner change and concurrent sexual partnerships, migration, and commercial sex, likely play a role. Whether infectiousness varies by virus subtype (subtype C is dominant in southern Africa) remains debated. Under the South African presidency of Thabo Mbeki, AIDS denialism (the view that HIV is not the cause of AIDS) led to delayed implementation of ART and resulted in thousands of deaths.

Understanding the emergence and origins of HIV/AIDS will provide insight into global vulnerability to new infectious diseases. Without globalization and its central characteristic of increased movement of people, HIV might have remained in central Africa and the AIDS pandemic might have been delayed or might not have occurred. Although commerce and trade are as old as civilization, international air travel increased greatly in the latter half of the 20th century and enabled people to arrive at their destinations in greater numbers and within the incubation periods of many infectious diseases. The prolonged period between HIV infection and symptomatic AIDS, ≈11 years in adults, allowed widespread HIV transmission before recognition of the epidemic and any prevention attempts.

The response to HIV/AIDS epitomizes a new concept of global health. Essentials of global health today are its integration of core public health attributes (data and surveillance-based approaches, emphasis on populations, goals of social justice and equity, and prioritization of prevention), expansion into new areas such as treatment and health systems, and focus on emerging challenges beyond traditional priorities. New areas of emphasis include health security, chronic diseases (e.g., diabetes), and road traffic injuries. Global health is now about how the world deals with health rather than how a particular country addresses health problems in other countries.

## AIDS and the Globalization of Science, Research, and Practice

A positive development in the response to AIDS has been its effect on science and the globalization of research and practice. Retrovirology and immunology became well-supported disciplines whose practitioners interacted productively with workers in other subjects such as epidemiology. Cohorts of physicians and scientists built their careers in basic as well as applied and clinical research. The frequency with which tuberculosis occurs in HIV-infected persons has led to a resurgence of interest in the diagnosis and treatment of this ancient disease, especially in Africa. Advances in the treatment of HIV-associated OIs have benefited other immunosuppressed persons. In addition, sexual and reproductive health gained renewed prominence.

Scientific advances resulted in the development of lifesaving, albeit not curative, treatment for HIV. Beginning with the approval of AZT (azidothymidine or zidovudine) in 1987, the development of antiretroviral drugs and the design of simple and standardized approaches for therapy in the developing world constituted a public health triumph. By the end of 2009, >5 million persons in low- and middle-income countries were accessing ART, unimaginable just a few years before and made possible through the use of generic drugs, price reductions for brand-name drugs, and efforts of international donors through initiatives such as the President’s Emergency Plan for AIDS Relief and the Global Fund.

Research on the prevention of mother-to-child transmission of HIV has led to interventions with the potential to virtually eliminate HIV disease in children. Screening of donated blood and plasma for HIV and heat treatment of blood products have virtually eliminated transfusion-related HIV in high-income countries and vastly reduced its occurrence throughout low- and middle-income settings. Research has identified viable options for HIV prevention in IDU, such as needle and syringe exchange and opioid substitution therapy. Hospital hygiene and safe injection practices, previously neglected in much of the developing world, have become topics of global concern.

The earliest international collaborative field investigations on HIV/AIDS were in 1983 in Rwanda and the former Zaire, now the Democratic Republic of Congo. In 1984, Projet SIDA (French for AIDS Project) was established. This project, a joint venture between CDC, the National Institutes of Health, the Belgian Institute of Tropical Medicine, Mama Yemo Hospital (Kinshasa), and the then Zairian Department of Public Health, conducted landmark epidemiologic studies in central Africa. At one time Zaire had the highest citation index for AIDS research in the world. Subsequently, a second CDC-sponsored field station, Projet Retro-CI, contributed to the body of research from in western Africa, documenting lower pathogenicity and transmission rates for HIV-2, which indicated that although HIV-2 was a cause of AIDS, it was unlikely to result in a pandemic.

Numerous other international collaborations on HIV/AIDS had influence far beyond research publications. Investigators from low- and middle-income countries were trained, university exchanges arranged, and numerous careers influenced and internationalized with incalculable effects. Many US and European universities established HIV training and research collaborations with their counterparts in developing countries. These relationships have built platforms upon which new initiatives, in areas such as maternal and child health, could be built. One of the lasting contributions of international HIV/AIDS work may be the training and empowering of professionals in low- and middle-income countries to influence health in their own countries.

Despite the advances in HIV prevention and treatment, the challenges remain daunting. In 1984, the US Secretary of Health and Human Services famously predicted the availability of an HIV vaccine within 2 years. Now, >25 years later, an effective vaccine remains elusive. Although billions of dollars have been expended on prevention research, an estimated 2.6 million persons acquire HIV annually. Only about a third of patients who qualify for treatment under the relatively conservative WHO guidelines actually receive it, and neither the optimal time for treatment initiation nor the optimal use of antiretroviral drugs to interrupt transmission have been determined. Tuberculosis remains a major killer of HIV-infected persons in Africa, our tools for combating it are outdated, and coordination between tuberculosis programs and HIV/AIDS programs remains less than optimal.

## The AIDS Response, Nothing for Us without Us

Activism and advocacy profoundly influenced the response to HIV/AIDS. Outside the gay community, initial concern about HIV/AIDS was largely limited to scientists tracking the epidemic or searching for a cause. In the face of stigma, discrimination, and indifference to their friends dying, affected communities organized to provide prevention advice, care, and support. Community groups like Gay Men’s Health Crisis sprung up, delivering services and engaging in political activities. Organizations such as ACT UP undertook acts of civil disobedience to influence the research agenda, improve access to HIV drugs, and lower the cost of treatment.

Although early activists were predominantly American MSM, their work influenced other affected communities. When the magnitude of the epidemic in Africa became apparent, activists from the Northern Hemisphere contributed to demands for treatment access in the Southern Hemisphere. Vulnerable groups, including sex workers and IDU, made themselves heard internationally in an unprecedented way. “Nothing for us without us” captures the insistence of affected communities that they participate in the design of programs and interventions.

A key figure in the global response was Jonathan Mann, an epidemiologist from CDC who served as the founding director of Projet SIDA and was appointed in 1986 as the first director of WHO’s HIV/AIDS program. Mann recognized that the global spread of HIV/AIDS represented unequal vulnerability more than it did individual behavior, and he defined human rights as central to health and an effective HIV/AIDS response. He clashed with WHO leadership and bureaucracy and resigned in 1990. Tragically, Mann died in a plane crash in 1998.

Much has been written about the different ways that HIV/AIDS has been addressed, compared with other sexually transmitted infections, and the term AIDS exceptionalism has been coined. For example, specific consent forms and counseling were required before HIV testing, and limits were placed on sharing patient names between health jurisdictions for HIV surveillance purposes. These practices responded to concerns of affected communities that infected persons would be subject to discrimination such as termination of insurance or employment. Mandatory HIV testing, unhelpful and discriminatory, was largely prevented, but exceptionalist views may also have delayed expansion of HIV testing in clinical settings and thus access to care, including in the Southern Hemisphere. As HIV became treatable and surveillance practices successfully protected confidentiality, much of the exceptional approach to HIV gradually diminished.

Major human rights challenges persist, however. IDU and MSM suffer intense discrimination in many countries; their prevention needs are neglected and their very lives are sometimes in danger. Gender inequalities remain a driver of ill health. Increased attempts at criminalizing HIV transmission and continued travel restrictions for HIV-infected persons illustrate the enduring relevance of Jonathan Mann’s message.

## AIDS and the Architecture of Global Health

HIV/AIDS played a major role in shaping current global health architecture. The threat posed by HIV led WHO to establish a dedicated program in 1986. In 1996, the Joint United Nations Programme on HIV/AIDS was established to coordinate the multisectoral response. In 2001, the United Nations General Assembly Special Session on HIV/AIDS, the first high-level summit ever devoted to a disease, committed the world to specific targets. In 2002, the Global Fund to Fight AIDS, Tuberculosis and Malaria was created, and a year later, US President G.W. Bush announced the President’s Emergency Plan for AIDS Relief, the largest bilateral health program ever undertaken. The scale-up of HIV/AIDS services has highlighted the need to focus on strengthening health systems and on other health-related Millennium Development Goals relating to maternal and child health.

The increase in actors in global health, including philanthropic organizations such as the Bill and Melinda Gates Foundation and the William J. Clinton Foundation, in part resulted from, but also coincided with, development of the global AIDS response. A result of the altered landscape is a diminution of the World Health Assembly’s influence on global decision making and WHO’s role in technical assistance. At the same time, HIV/AIDS demonstrated WHO’s unrivaled convening authority and the influence of its normative guidelines for global practice, such as for HIV/AIDS treatment. Major contributions from the Joint United Nations Programme on HIV/AIDS include global prioritization of HIV/AIDS and resource mobilization, epidemiologic monitoring, advocacy for treatment in low-income settings, and promotion of sound policies.

A problem with the early response in the United States as well as globally was an overemphasis on universal vulnerability, the concept that everyone is at risk. Predictions of widespread, generalized HIV/AIDS epidemics among heterosexual persons outside Africa, especially in Asia, were not borne out. The concept of “know your epidemic,” highlighting the need to focus interventions where HIV transmission is most intense, came surprisingly late, along with acknowledgment of the fundamentally different nature of the epidemic in sub-Saharan Africa compared with elsewhere.

Many countries with concentrated epidemics have had difficulty accepting that communities of MSM and IDU existed in their midst, let alone mounting targeted responses. Vigilance is required to ensure that resources are deployed to the right places in a timely fashion, rather than to general population groups that are politically safer but at lower risk. Characteristically, it has taken AIDS to bring the existence of marginalized groups such as sexual minorities to attention in low- and middle-income countries and to highlight their vulnerability and needs.

## AIDS and the Future

We should not expect a single leader or intervention to deliver an abrupt end to the HIV/AIDS pandemic, yet the tide can be turned with principled pragmatism, adequate resources, trust in communities, and science as our guide. At times the process is slow. For example, US government support for needle and syringe exchange to prevent HIV in IDU did not happen until Barack Obama became US President (2009), and scale-up of male circumcision has been inadequate. But a middle way has to be found between arguments for the magic bullet of the moment and calls for unrealistic social and behavioral change with regard to sex and drug use.

We (the authors) have 4 priorities: 1) defining the best ways to use existing interventions to interrupt HIV transmission, 2) continuing the focused search for new knowledge and interventions, 3) resolving how best to use HIV testing and antiretroviral drugs for prevention as well as treatment, and 4) ensuring sustainability and commitment for the global response. Aspirations for social justice, human rights, and decency must motivate the response while epidemiology and surveillance provide technical direction as well as evaluation. True country and community ownership of the response is essential because solutions wanted more by donors or governments than by affected communities themselves almost never succeed.

Further success in HIV/AIDS prevention and treatment is challenged by numerous threats including fatigue and shifting priorities on the part of donors, the global financial downturn, and diversion of attention to other health problems plaguing the developing world. Better integration of HIV/AIDS efforts and interventions with those addressing maternal and child health are needed, and the global health infrastructure supported by HIV/AIDS scale-up will have to face the looming pandemic of noncommunicable diseases. Regardless how global health evolves, the unfinished agenda of HIV/AIDS must remain central.

## Conclusions

Although we continue to face many challenges while responding to HIV/AIDS, we must also acknowledge the enormous scientific, social, and human achievements of the past 3 decades. The epidemic has severely tested many countries, especially those with the most limited resources, yet these countries have generally responded with decency, compassion, and good judgment. Despite the human and financial costs, millions of infections have been prevented and millions of life-years saved. The response to AIDS will be a benchmark against which responses to future health threats will be compared.

Many themes of the HIV/AIDS epidemic were captured by Albert Camus in his classic novel The Plague*,* and the expectations expressed therein largely apply. Inevitably, the story of HIV/AIDS “could not be one of final victory. It could be only the record of what had to be done, and what assuredly would have to be done again in the never-ending fight against terror and its relentless onslaughts.” An enduring frustration is that we will not know how the story of AIDS will finally end because the epidemic will outlast us. A perpetual challenge will be living up to the commitment and courage of those who went before—health workers, scientists, and affected persons—who faced the unknown and took risks. In general, 30 years of AIDS confirm that there is indeed “more to admire in men than to despise.” And while the epidemic continues, the world of global health has changed for the better.

